# Technology‐Facilitated Sexual Abuse and Exploitation of Children Is Everyone's Problem; How Can Healthcare Practitioners Respond to the Second Global Pandemic of the 21st Century? A Narrative Review

**DOI:** 10.1111/jpc.70148

**Published:** 2025-07-20

**Authors:** Joanna Tully, Susan McLean, Janine Rowse

**Affiliations:** ^1^ Victorian Forensic Paediatric Medical Service Melbourne Australia; ^2^ Department of Forensic Medicine Monash University Melbourne Australia; ^3^ CyberSafety Solutions Melbourne Australia

**Keywords:** child sexual abuse, healthcare, prevention, social media, technology

## Abstract

Sexual harm of children online is commonplace. The proposed ban on social media for children under 16 in Australia has been met with mixed responses about its likely effectiveness in preventing harm, as well as the possible negative effect on caregiver vigilance and children's willingness to report. Consideration of the possibility of online sexual harm may now be even more important, and healthcare practitioners are well placed in their interactions with children and caregivers to provide advice and recognise and respond to concerns. This article explores the spectrum of Technology‐Facilitated Child Sexual Abuse and Exploitation (TFCSAE) and provides guidance for practitioners, including around recognising alerting signs, useful tips for caregivers, a suggested framework for conversations with children, as well as including helpful resources for children and families. The need for the development of specific training for healthcare practitioners in Australia in relation to the recognition and response to TFCSAE is discussed.

**Summary**

- A call for action, the healthcare response to TFCSAE
  ○ Consider online harm.
  ○ Ask about online safety, every parent, every child, every consult.
  ○ Be curious, know the signs, ask the questions, know how to respond.
- Advise caregivers:
  ○ Young children do not need a smartphone; give them a feature phone.
  ○ No devices in bedrooms or bathrooms. Use and charge in shared spaces.
  ○ Instal parental controls and supervise children online.
  ○ Do not threaten to confiscate devices, do not blame children. Communicate, educate, support, and empower children to be safe online.
  ○ Create a contract with your child about online use.
  ○ Stay educated about evolving online risks and ways to keep your child safe https://www.esafety.gov.au/, https://www.esafety.gov.au/parents/issues‐and‐advice/protecting‐children‐from‐sexual‐abuse‐online. https://www.esafety.gov.au/sites/default/files/2024‐06/Every‐online‐safety‐conversation‐matters.
- Educate children:
  ○ An online friend is a real‐life stranger. Do not connect with people you do not know.
  ○ ‘It's OK to say no’ to sharing intimate images.
  ○ Online sexual abuse is never your fault.

## 1 Introduction

Young people are experiencing high rates of mental health difficulties and use of the internet, particularly social media, has been suggested to be a factor [1, 2]. This has been the catalyst for the introduction and passing of the Online Safety Amendment (Social Media Minimum Age) Bill 2024, to prevent Australian children under 16 years accessing social media [3]. However, reactions to this approach to improving online safety have been mixed [4, 5, 6, 7, 8]. Technology is inherent in contemporary society and provides significant benefits to children in terms of social connectivity and learning [6]. A cybersystem is arguably part of a child's ecology [9, 10] and this strongly suggests that digital literacy education in conjunction with creating systems that prevent access to *harmful* material by systemic regulation (such as the digital duty of care legislation) is a more sustainable approach. A blanket ban will be difficult to enforce, may not include sites known to contain harmful content, restrict children's access to beneficial material, disempower children to navigate online spaces safely, increase caregiver complacency, and create further barriers to children disclosing negative online experiences. A ban may also inadvertently increase the risk of sexual harm from perpetrators exploiting children's fear of the negative consequences of disclosure, and platforms being disincentivised to prioritise child safety features [6, 8, 11, 12]. Until the downstream ramifications of legislative reform become apparent, it is paramount that professionals be vigilant to the possibility of online harm, recognise the signs, and know how to respond.

## 2 What Is Technology‐Facilitated Child Sexual Abuse and Exploitation (TFCSAE)?

TFCSAE is the umbrella term used to describe a range of sexually abusive and exploitative behaviours that occur online or are facilitated through the use of information and communication technologies [13].

**FIGURE 1:**
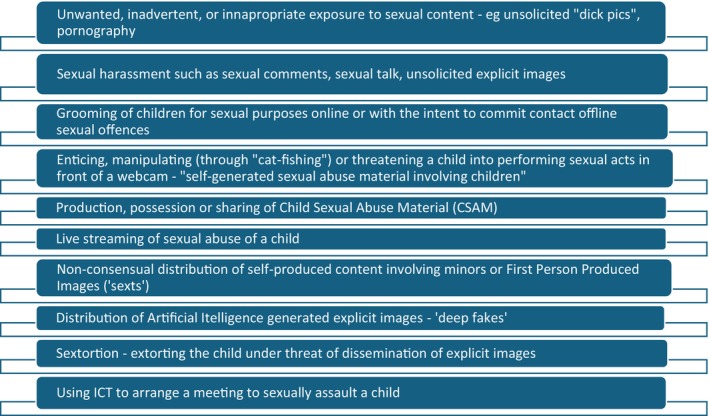
The spectrum of TFCSAE. Prevalence rates vary across types, with an overall global prevalence of around 15% [14].

There are a range of ways in which children can be sexually harmed through use of technology (see Figure 1).

## 3 Why Should We Be Concerned? The Global Paediatric Pandemic

The number of children being groomed and sexually harmed online is staggering. A 2024 report by Childlight estimated that up to 300 million children each year experience sexual abuse or exploitation online [15]. Over 7% of Australian men surveyed reported that they have engaged in online behaviours toward a child at some point in their lifetime that could be considered sexually abusive [15]. The appetite for Child Sexual Abuse Material (CSAM, previously termed child pornography) is high, driving demand and creating a multibillion‐dollar industry [16]. Between 2018 and 2023, 11% of over 110 million searches on four popular Tor search engines were for CSAM [17] and almost two‐thirds of the millions of sexual images of children circulating online depict prepubescent children [18] including 5% of CSAM‐related Tor engine searches being for material involving babies and toddlers [17]. When all forms of TFCSAE are considered, one in eight children are estimated to be a victim [14, 15].

## 4 ‘Yes, But Not My Child …’

Most Australian adolescents and many primary school aged children have a smartphone; devices small enough to fit in a pocket or under a doona. While the internet is a place where young people connect, create relationships, explore cultures and ideas, and feel accepted as part of a community that may not exist in their offline world, it is also a place where it is easy for individuals to isolate, groom, exploit, and manipulate children for sexual purposes. The internet is now the dominant way of adults establishing sexual contact with children and online sexual experiences with unknown adults are very common [19, 20]. Children are routinely being contacted by adult offenders, sometimes by many at any one time, on popular platforms, apps and games that parents perceive to be safe [9]. Despite the mainstream appeal of platforms such as Instagram, Snapchat and Tik Tok, it is these widely popular apps that currently pose the greatest risk to children [20]. While the minimum age required to use most social media platforms is 13, and to use dating apps and adult content sites is 18, age‐verification technology is either absent or woefully inadequate and children are regularly accessing these platforms, often with parental assistance. A large representative survey conducted in the United States found that one in four boys aged 9–12 years had used a dating app [20]. The Australian Institute of Criminology examined data on Australian adult dating platform users, finding 9% of respondents reported they had used a dating app when they were a child with 59% reporting they had received sexually explicit requests from an adult who knew they were under 18, including being offered payment for explicit images [21].

**FIGURE 2:**
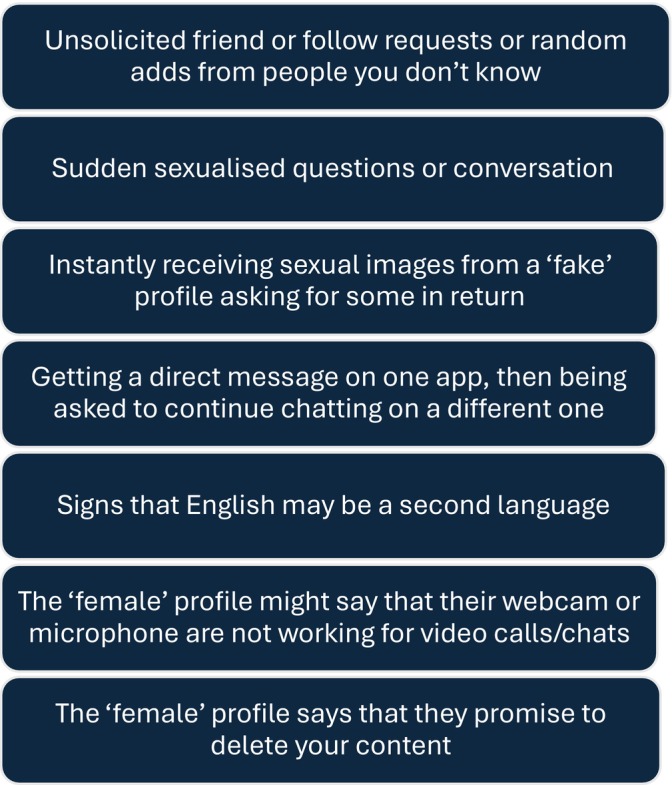
Warning signs signalling possible sextortion attempt.

There are more contemporary forms of TFCSAE that warrant highlighting and that have garnered recent attention. Live‐streamed or ‘on‐demand’ CSAM is transmitted to the viewer through streaming over the internet in real time. The perpetrator may have coerced, blackmailed or threatened the child into performing sexual acts on camera, often after successfully obtaining a sexual image from the child, or the child may be willingly ‘performing’ in the mistaken belief that they are romantically attached to a person who is, in reality, a child sex offender. This self‐generated CSAM is often produced in a child's bedroom or household space [22, 23]. Sextortion has been described as the greatest contemporary cyberthreat to children with more victims per offender than any other type of child sexual exploitation offence [24] and Australia has one of the highest prevalences [25]. Sextortion is blackmail using intimate images to extort sexual favours, money or other benefits under the threat of wide dissemination of the material [25] and begins with a young person, often male, receiving communication from an unknown sexually attractive ‘peer’ whom they enthusiastically ‘friend’ and with whom they develop a rapid online relationship. Once the young person has been persuaded to share an explicit image, there is a sudden and rapid escalation of threats and intimidation. Sharing of self‐made sexually explicit images (First Person Produced Images or ‘sexts’) is now commonplace [9] and this provides fertile ground for organised crime syndicates to exploit. Children should be taught the warning signs (see Figure 2).

Children are also at risk of offline contact sexual offences facilitated through online activity. There is a lack of traditional ‘stranger danger’ created by the online communication prior to meeting in person known as the online disinhibition effect [26]. In an Australian study of sexually assaulted adolescents undergoing forensic medical examination who met their offender online, the sexual assault most often occurred on the first face‐toto‐face meeting following an often prolonged period of online communication, exemplifying the power of the online disinhibition effect [27].

Parents understand the potential for sexual harm online but are largely unaware of the scope of the problem or level of risk [28] and fail to appreciate that it could happen under their roof—‘yes, but not my child’. TFCSAE can happen to any child, anytime, anywhere.

## 5 Why Does It Matter? The Downstream Harms of TFCSEA

Victims of TFCSAE experience persistent mental health problems including feelings of shame, worthlessness, depression, anxiety, disordered eating and sleeping, posttraumatic stress disorder, self‐harm and relationship difficulties [29, 30, 31, 32, 33]. Victims feel intense guilt and shame over their perceived complicity in the abuse, and that they deserve the consequences [9, 33]. Contrasting to contact‐only sexual offences, the persistence of the images online over time and the vast reach of the material leads to unprecedented re‐victimisation, constant fear of the images being discovered, and an inability to find closure [29, 32, 33, 34, 35]. Victims describe intense distress at knowing that explicit images of themselves might be used for the ongoing gratification of countless individuals, speaking of their fear of being recognised [9, 33, 34]. Importantly, a child who is recognisable in explicit material created using generative Artificial Intelligence (‘deepfakes’) may experience the same trauma and sense of revictimization [22, 36].

## 6 Why Don't They Tell? Understanding Barriers to Disclosure

Sexual assault is an under‐reported crime and there are additional barriers to reporting technology‐facilitated sexual crimes, victims being described as ‘the children who do not tell and the children who cannot tell’ [37]. The stigma and shame inherent in the child's perceived role in the abuse is significant. Children may have lied about their age, fear the reaction of their family, or not trust that adults will believe them, or know how to protect them. The online disinhibition effect results in an enhanced sense of friendship and trust, with victims often describing a profound intimate connection to their online ‘friend’, not recognising the interactions as abusive [19, 38]. The legislative reform regarding social media access may create further barriers to disclosure due to the young person's perception of the consequences of authorities discovering they accessed a banned platform.

Children seem more likely to disclose negative online sexual experiences to their peers than to a parent [35] and one important reason for this, unique to child victims, is fearing intense negative reactions from parents, particularly blame and punitive monitoring or removal of their device to which young people describe being emotionally wedded; ‘my phone is my life’ [35, 39]. Disclosure often takes place over time [35, 38] and may occur more readily if an adult asks directly about negative experiences in a warm, open environment [40]. The response to a disclosure should include addressing the fear of negative consequences, actively listening to and believing the child, and minimising guilt and shame [35, 41]. Children need to be reassured that TFSEA is never their fault.

## 7 Assisting Disclosure—What Can Healthcare Providers Do?

TFCSAE presents a unique and rapidly evolving challenge for professionals wishing to allow children their autonomy, empower them to make informed choices, and learn to safely navigate the digital world while protecting them from exploitation and harm. Healthcare providers, invested in the health and wellbeing of children, are well placed to recognise and respond to concerns about online harm, but report being technologically out of touch, lacking in knowledge and understanding about the online practices of young people [9, 10, 16, 29, 31], and poorly equipped to respond to concerns about online harm [9, 10, 42, 43, 44].

Health professionals can play a key role in the response to TFCSAE. They can support caregivers and raise awareness of the potential for harm, directing them to useful resources. They can reduce shame associated with negative online experiences for young people by normalising conversations around online activity and potential harms, establish trust and ask questions to facilitate disclosure, recognise alerting signs, know how to respond appropriately, and support reporting and recovery. The benefit of practitioners asking questions about online activity is acknowledged [45, 46]; however, there is a lack of operational definition, clinical training and available assessment tools in relation to identification and response to TFCSAE to support practitioners [9, 31, 38, 42, 43]. Existing training opportunities, mostly UK‐based, are neither standardised nor mandatory [38, 47] and existing assessment tools are used ad hoc and are not always fit for purpose [38, 43, 44]. Health professionals do not frequently or routinely ask children about online experiences, expressing a lack of training and guidance around how to conduct conversations about online use and a lack of confidence in recognising signs and responding appropriately to disclosures [9, 16, 42] despite young people agreeing that health practitioners should contribute to online safety [45].

**FIGURE 3:**
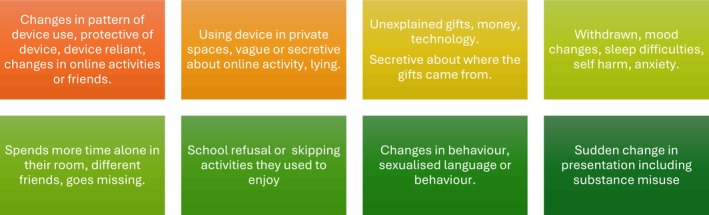
Signs that may alert a practitioner to TFCSAE.

Victims of TFCSAE may present to medical providers from a range of specialities, for a range of reasons, and with a range of signs and symptoms, either directly related to the abuse or arising from unrelated medical issues. All health providers interfacing with children need to acknowledge the profound effect that TFCSAE may have on children's physical and mental health and be familiar with alerting signs that may vary according to the age and developmental stage of the child or may not be present at all [16, 22] (see Figure 3). Children with communication difficulties, intellectual disability, mental illness and those without a supportive home environment, such as those in state care or those whose parents are emotionally unavailable for a range of reasons, are more vulnerable and less likely to disclose [35, 48]. A high degree of vigilance is necessary.

### 7.1 Healthcare Providers Should Support Caregivers

The online safety of children is a prime responsibility of caregivers and the social media ban risks complacency, due to the false assumption that sources of online harm are no longer accessible. Health professionals should continue to raise awareness, educate, and provide resources to support parents. During medical encounters parents should be routinely asked about their child's online activity and safety whenever appropriate to do so. Limiting and controlling online access is generally less effective in preventing harm than discussion and mediation, indicating that encouraging caregivers to have open conversations with their child is important. Creating a written contract around online use may be helpful [49, 50]. The need to instal adequate parental controls and to supervise children online needs reinforcing. Advising parents that devices should not be allowed in bedrooms or bathrooms and should be charged in shared spaces overnight provides an unambiguous message. Children need reassurance that devices will not be confiscated, but that they will be supported if they disclose inappropriate interactions. Children of primary school age do not generally need a smart phone, and parents need to carefully consider the age at which they will allow their child a smart device. Caregivers can be directed to https://www.esafety.gov.au/parents/issues‐and‐advice/protecting‐children‐from‐sexual‐abuse‐online for tips and resources to keep their child safe, and www.thinkyouknow.org.au to learn more about online exploitation. Practitioners should consider providing parents with the excellent ‘Every Online Safety Conversation Matters’ fact sheet accessible at www.esafety.gov.au/everyonline [51].

### 7.2 Healthcare Providers Should Talk to Children

Biddle engaged youth when determining good practice indicators to guide practitioners as to when, how, and what should be discussed during consultations, concluding that conversations regarding online activities should be initiated at first and subsequent assessments, particularly for certain ‘red flag’ presenting problems (see Figure 3), and that this encouraged normalisation, reflection, and self‐awareness [45]. Conversations were more effective if introduced spontaneously, were open‐ended, contextualised, and demonstrated curiosity about the child's cyberlife, while explicitly addressing fear of judgement and limits to confidentiality. ‘Tick‐box’ lists were judged ineffectual [45]. Talking about positive aspects of the child's online experiences prior to addressing the negative is good practice, as well as acknowledging how commonplace online harm can be. Conversations with children could be structured focusing on their chosen activities and preferred content, their patterns of use, online experiences (positive and negative), and specific topics such as image creation and sharing [45] (see Table 1).

**TABLE 1:** Suggested structure for conversations with children around online activity.

**Set the scene**	Open and nonjudgmental. Speak to child alone where possible. Discuss confidentiality limits. Ensure young person knows that questions are routine.
**Contextualise**	Be curious. Tell the young person that you know how common online harm is and that you wish to ensure their safety and support them.
**Enquire about positive experiences—**activities and content viewed.	Acknowledge benefits. Enquire about activities, content, and patterns of use eg ‘Tell me what you enjoy doing online? What are your favourite apps/sites? Who do you talk to online? What do you post? Do you have romantic relationships online?’ Focus on the ‘which’ and ‘how’, not the ‘whether’.
**Enquire about negative experiences—**what they encounter rather than what they have done.	Address fear of judgement or blame. Offer help and support. Acknowledge that negative things happen online that create anxiety, fear, or shame. ‘Has anything bad, sad, or scary happened to you online? Have you ever seen anything online that has upset or frightened you? Is anyone upsetting you online? Is it happening right now?’
**Address specifics if negative experiences disclosed**	Questions about specific sites/apps used, content created, participation in online groups. Response of child to date.
**Provide support**	Talk to caregiver if child less than 12 or risk high. Who does the child trust and who can they talk to? How can they keep themselves safe? Advise about reporting to e‐safety/ACCCE/police.
**Encourage reflection**	Taking control of online safety, use of online tools, self‐imposed limits and breaks, unfollow accounts that are negative, understanding algorithms, privacy settings. Young people can be directed to www.eSafety.gov.au/kids if under 12 and www.eSafety.gov.au/young‐people if older.

All children, including primary school aged children, should be asked about online activity whenever possible, but particularly if they present with mental health or mood concerns, school refusal, behaviour change, somatisation or substance use. The HEEADSSS screening tool for adolescents includes enquiries about online safety (S = safety) and should serve as a reminder when speaking to children over 12. Training opportunities, such as The Path to Protection course, could be considered by professionals (https://www.mariecollinsfoundation.org.uk/What‐We‐Do/Training).

The response to a disclosure should be calm, met with firm statements of belief and absence of blame. The child should immediately cease contact with the perpetrator and screenshot all communications. The child may need help talking to their caregiver, who may need support to minimise intense reactions or negative responses to the child, particularly blame and threats to remove their device. If the child is in immediate danger, call 000 to report to Police. Be aware of local mandatory reporting requirements. TFCSAE can also be reported over the counter at any Police station, and sextortion involving a child under 18 years can be reported to accce.gov.au/report, option 4. The child or family may need assistance with reporting. Excellent resources are available through the website of the e‐safety commissioner and Emerging Minds in relation to this. An anonymous report can be made through e‐safety, who can work to remove online material, and this can be very reassuring to the young person (www.eSafety.gov.au/report) and the TakeItDown tool (https://takeitdown.ncmec.org/) provides a secure, anonymous way to prevent sexual images or videos being uploaded and shared on online platforms. The ACCCE website lists a range of counselling and support services for children and families.

## 8 Conclusion

Tackling TFCSAE requires a societal response. While we applaud the spotlight on the issue of children's online safety afforded by the proposed social media ban, ensuring and sustaining children's safety online requires a broader raft of measures that includes empowerment of children through delivery of early, coordinated, mandated digital literacy education as a core subject in schools, as well as legislating to ensure tech companies prioritise, and are accountable for, children's safety. While healthcare professionals have an important role to play, they feel poorly equipped to do so. The development of standardised and specific practice tools for use during assessments of children, as well as provision of consistent training for healthcare providers in Australia, needs consideration. In the meantime, healthcare professionals, well placed in their frontline interactions with children, can start the conversations to, once again, rise to the challenge of a global pandemic.

## Author Contributions

J.T. conceptualised and drafted the manuscript. S.M. provided content expertise and factual review and contributed to manuscript revisions. J.R. contributed to conceptualisation of the manuscript and to manuscript revisions.

## Conflicts of Interest

The authors declare no conflicts of interest.
